# Pig movements in France: Designing network models fitting the transmission route of pathogens

**DOI:** 10.1371/journal.pone.0185858

**Published:** 2017-10-19

**Authors:** Morgane Salines, Mathieu Andraud, Nicolas Rose

**Affiliations:** 1 ANSES–Ploufragan-Plouzané Laboratory, Ploufragan, France; 2 Université Bretagne-Loire, Rennes, France; University of Bristol, UNITED KINGDOM

## Abstract

Pathogen spread between farms results from interaction between the epidemiological characteristics of infectious agents, such as transmission route, and the contact structure between holdings. The objective of our study was to design network models of pig movements matching with epidemiological features of pathogens. Our first model represents the transmission of infectious diseases between farms only through the introduction of animals to holdings (Animal Introduction Model AIM), whereas the second one also accounts for pathogen spread through intermediate transit of trucks through farms even without any animal unloading (i.e. indirect transmission–Transit Model TM). To take the pyramidal organisation of pig production into consideration, these networks were studied at three different scales: the whole network and two subnetworks containing only breeding or production farms. The two models were applied to pig movement data recorded in France from June 2012 to December 2014. For each type of model, we calculated network descriptive statistics, looked for weakly/strongly connected components (WCCs/SCCs) and communities, and analysed temporal patterns. Whatever the model, the network exhibited scale-free and small-world topologies. Differences in centrality values between the two models showed that nucleus, multiplication and post-weaning farms played a key role in the spread of diseases transmitted exclusively by the introduction of infected animals, whereas farrowing and farrow-to-finish herds appeared more vulnerable to the introduction of infectious diseases through indirect contacts. The second network was less fragmented than the first one, a giant SCC being detected. The topology of network communities also varied with modelling assumptions: in the first approach, a huge geographically dispersed community was found, whereas the second model highlighted several small geographically clustered communities. These results underline the relevance of developing network models corresponding to pathogen features (e.g. their transmission route), and the need to target specific types of holdings/areas for surveillance depending on the epidemiological context.

## 1. Introduction

Swine infectious diseases have economic consequences for the pig industry and can affect public health. They can be transmitted from farm to farm through animal trade, either because of the introduction of infected animals, or only because of transit movements of contaminated trucks acting as mechanical vectors [1]. Disease spread is closely linked to the movement network topology [1, 2]; gaining insights into spatial and contact patterns of pig trade could therefore be a major lever to control the spread of swine infectious diseases. To do so, animal movement data are increasingly modelled into networks and studied using social network analysis (SNA) methods [2–22]. Animal trade networks are composed of nodes, which are either farms or slaughterhouses, markets, trade operators, etc., and of links, which are shipments of animals between these units. These networks are directed: animal movements along the network links are considered directed paths for the spread of a disease from one farm to another. Cattle, sheep, pig and poultry markets have already been modelled in several countries [2, 4–22], using either movements reported by farmers through questionnaires, or movements systematically recorded in a harmonised database. Unlike cattle movements, a special feature of swine trade data is that pig movements are reported at a batch scale, without the possibility of tracking animals individually. Moreover, the pig production sector is organised in a pyramidal way, with movements going from the nucleus and multiplying farms at the top, to the production farms at the bottom (from farrowers to finishers). This particular structure affects the network topology and has to be accounted for [6]. Pig movements can exhibit intricate patterns, for instance when trucks collect pigs at several farms before unloading all of them at a single site (e.g. a slaughterhouse). To our knowledge, most of swine trade networks published in the literature have simplified these complex trajectories going through several farms by representing only direct operations from the loading locations to the unloading sites [2, 7–10, 19, 23]. By doing so, intermediate transit movements of trucks in farms without any animal unloading have been neglected. Yet these movements can contribute to the spread of diseases for which indirect transmission through mechanical vectors occurs (e.g. African Swine Fever—ASF, Porcine Epidemic Diarrhoea—PED, Foot and Mouth Disease–FMD, Porcine Reproductive and Respiratory Syndrome Virus—PRRSV) [24–26]. To fit as closely as possible with the pathogens’ epidemiological features, network models should take their various transmission routes into account. To explore the role of trucks in indirect disease spread, some research teams developed two-mode networks with trucks or rounds being considered as a second class of nodes in addition to holdings [6, 21]. This method makes it possible to obtain relevant data regarding the functioning of rounds, such as the number of rounds concerning a given farm, or the number of holdings connected in a round. However, two-mode networks are not easy to analyse: centrality measures cannot all be computed, contact chains are not calculated, and communities and connected components are usually not looked for [6, 21]. Two-mode networks are thus often altered in a one-mode network to be more deeply analysed [6].

The objective of our study was therefore to design two one-mode network models matching with the transmission route of pathogens, and to analyse empirical data of French pig trade. We focused our model analysis on the different levels of the pyramidal structure inherent to the pig production system.

## 2. Materials and methods

### 2.1. Data

#### 2.1.1. Database description

Since 2010, pig movements in France have been recorded and stored in the National Swine Identification Database (BDporc). This database is managed by swine industry professionals and is recognised by the French Ministry for Agriculture. For the present study, we analysed the data from June 2012 to December 2014. Two levels of information were gathered in the dataset: the characteristics of swine production units and the details of the animal movements between the different production sites. The main features of all swine holdings in mainland France are included in the database: identification number, type of holding (farm, slaughterhouse, rendering company, market, assembly centre, trading company), type of farming activity (boar station *BS*, nucleus *SEL*, multiplication *MU*, farrowing *FA*, farrowing-to-finishing *FF*, finishing *FI*, farrowing-post-weaning *FPW*, post-weaning *PW*, post-weaning-finishing *PWF*, small producers *SP*), type of production (free-range or not), and location (post code and GPS coordinates). Movements of pigs were reported at a batch level: groups of animals were sent off the production sites (loadings, further denoted L) and dispatched to either alternative production units or slaughterhouses (unloadings, further denoted U). A single truck could load and unload animals at several production sites: one round corresponds to a series of movements of a truck, from the first loading operation to the last unloading event making the truck empty. Each loading and unloading operation was individually reported for each round with several pieces of information: the farm and the round IDs, the chronological sequence of the operations during the round, the batch size and the animal category (breeding animals, piglets, and growing pigs).

#### 2.1.2 Data cleaning and pre-processing

Data included both movements occurring within France and movements from/to foreign countries. However, imports and exports of animals were recorded at the country level, with a lower data resolution than movements occurring within France. Therefore, movements from/to foreign countries were considered separately to have a global overview of international trade movements, when a thorough analysis of within-France data was performed.

A series of cleaning processes were performed on the dataset, discarding records for which the principal pieces of information were unavailable (e.g. round or herd identification numbers, animal category). Farms were categorised into 11 groups according to their major activity; markets, assembly centres and trading companies were gathered into the single “trade operators” category. Direct movements to slaughterhouses and rendering plants were excluded from the analysis as they do not play a major role in pathogen spread. When these movements were part of longer rounds collecting pigs from several herds before going to the slaughterhouse/rendering plant, only the last movement (from the last farm to the slaughterhouse) was excluded. Considering the absence of any seasonality in pig trade shown in previous studies [2, 7, 23, 27, 28], movement data were aggregated on a six-month basis.

### 2.2. Model design

**Fig 1:**
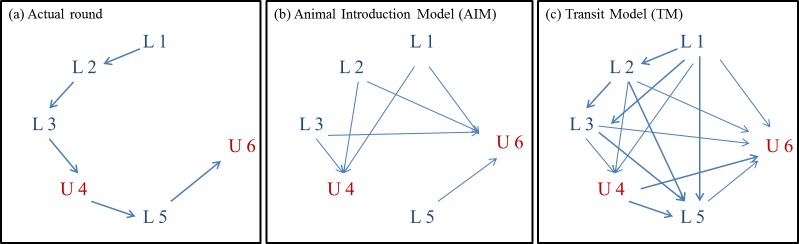
Types of network models built to represent pig movements. Nodes L and U correspond to holdings where loading and unloading operations occurred, respectively. The number corresponds to the chronology of animal collection by a truck in one round. Fig 1.a describes the actual round of a given truck, whereas Fig 1.b and Fig 1.c describes how the links between holdings were modelled, depending on the transmission route of the pathogen considered. In the Animal Introduction Model—AIM (Fig 1.b), movements forming a round were replaced with direct movements between holdings, i.e. intermediate transit movements of a truck through a farm without unloading any animal were neglected. This network accounts for the transmission of a disease only through the introduction of animals into farms. In the Transit Model—TM (Fig 1.c), each holding was assumed to be linked to every other upstream and downstream farm in a given round through incoming and outgoing links, respectively. This type of network can be used to explore the spread of a pathogen both through the introduction of animals to farms and through the indirect route.

One-mode directed networks were built: holdings were considered as nodes, movements between two nodes were considered as links. All movements between two given holdings during the time period were aggregated into a single link. We designed two types of network to model a round (Fig 1A) in two different ways depending on the route of transmission of the considered pathogen. *(i)* In the first network model, called hereafter the Animal Introduction Model (AIM) (Fig 1B), links between holdings represented movements of animals being unloaded at farms. In-between movements forming a round were replaced by direct movements between holdings, i.e. intermediate transit movements of a truck through a farm without unloading any animal were excluded. All sites corresponding to unloading operations were assumed to be linked to all prior loading sites of the same round. For example, assuming successive loadings at sites L1 and L2 followed by an unloading operation at site U4, then holding U4 was linked to L1 and L2. This model is relevant for pathogens that spread between holdings only through the introduction of animals to farms (i.e. diseases that spread via physical contact and for which the indirect transmission route is negligible). *(ii)* In the second network model, further denoted Transit Model (TM) (Fig 1C.), links between holdings represented both movements of animals and truck transit through a farm without any animal unloading. In a given round, each holding was therefore linked to all upstream and downstream farms (incoming and outgoing links, respectively). In other words, each round was modelled as a full graph. This model could be used for pathogens that spread not only because of the introduction of animals to farms but also through the transit of trucks through farms even without any animal introduction (i.e. diseases for which indirect transmission occurs, with trucks acting as mechanical vectors).

### 2.3. Network analysis

Considering the pyramidal structure of the pig production sector, all analyses presented below were performed at three different scales: the whole network, the breeding farm subnetwork (boar stations, nucleus/multiplication farms) and the production farm subnetwork. Network analysis was performed on within-France movements only.

#### 2.3.1. Network descriptive indicators

Several descriptive statistics of the network characteristics were calculated for each network model and for each semester to analyse changes in network properties over the study period. The first semester was running from January 1^st^ to June 30^th^, the second one from July 1^st^ to December 31^st^. The classical metrics that were computed were: the *size* (number of active nodes and links), the *average degree* (mean of the total number of ingoing and outgoing links for each node), the *average path length* (the average number of links along the shortest paths–or geodesics–between all pairs of nodes), the *diameter* (the longest geodesic), and the *density* (ratio of the number of links and the number of possible links for active nodes). We also calculated the *clustering coefficient* (proportion of neighbours of a node that are linked to each other), the *Jaccard similarity coefficient* (the JSC of two nodes being the number of common neighbours divided by the number of neighbours of each of the two nodes considered), the *assortativity degree* (Pearson correlation coefficient between the degrees of linked nodes), and the *reciprocity ratio* (proportion of mutual connections, in a directed graph). The distributions of the four main centrality measurements were computed for each holding type: *degree*, *in-degree* (number of different holdings from which a holding receives animals), *out-degree* (number of links going from a node), *closeness* (number of steps required to access every other node from a given node) and *betweenness* centralities (number of geodesics going through a node). For each network model, a power-law distribution defined as *p*(*x*)∼*x*^*α*^ was fitted to the observed degree distribution. We used a maximum-likelihood estimator to estimate scaling parameter (*α*) and the Kolmogorov–Smirnov (KS) goodness-of-fit statistic to test power law fit of the data as described by Clauset et al. [29].

#### 2.3.2. Detection of connected components and communities

**Connected components.** *Weakly connected components* (WCCs) are sections of the network where every holding can be reached from every other holding whatever the link direction. Based on this definition, no connection exists between two WCCs and they can be considered as independent subnetworks. *Strongly connected components* (SCCs) are subgraphs in which every node can be reached from every other node via one or several directed paths. The number of WCCs and SCCs and the size of the largest WCCs and SCCs were determined with the two network models AIM and TM, and for the whole population as well as separately for the breeding farm and production farm subpopulations.

**Communities.** Detection of *network communities*, defined as subsets of nodes in which there are significantly more links than expected by chance, i.e. groups of highly connected farms, was performed using the Infomap algorithm [30]. Briefly, the hierarchical map equation measures the per-step average code length necessary to describe a random walker’s movement on a network, given a hierarchical network partition, and looks for the community structure that minimises the expected description length of the random walker trajectory. In the core algorithm, each node is first assigned to its own module. Then, in random sequential order, each node is moved to the neighbouring module that results in the largest decrease of the map equation. When adding movements does not result in a decrease of the map equation, the node stays in its original module. This procedure is repeated, each time in a new random sequential order, until no move generates a decrease of the map equation. The network is then rebuilt, with the modules of the last level forming the nodes at this level, and, exactly as at the previous level, the nodes are joined into modules. This hierarchical rebuilding of the network is repeated until the map equation cannot be reduced further. The Infomap algorithm is the only one that can be applied on directed networks and it is considered to have the best performance [31]. We ran the algorithm with 1,000 trials, on the two network models AIM and TM. Like for the connected component detection, we looked for communities in the whole graph and in the two subgraphs (breeding/production farms). We also calculated the percentages of links connecting two different communities (i.e. bridges, or crossing links).

#### 2.3.3. Temporal network analysis

**Link and node preservation.** We counted the number of nodes remaining active from one semester to another, as well as the number of links being preserved from one semester to another.

**Node loyalty.** In order to explore the nodes’ tendency to re-establish connections with the same herds or to change trade partners over time, the *node loyalty* was computed for each kind of model. The loyalty measures the fraction of preserved links of a node for a pair of two consecutive network configurations in time, the time window in our case being a semester. It involves values between 0 and 1, a loyalty value of zero indicating that all connections were different between the two time windows, a loyalty of one indicating that exactly the same set of links was preserved. We computed the loyalty on the incoming contacts of nodes, thus quantifying the tendency of a farmer to purchase animals from the same sellers.

**Outgoing and ingoing contact chains.** The *outgoing and ingoing contact chains* (OCC and ICC, respectively) were computed for each type of holding over a one-month period. These measures capture the sequence of contacts through direct and indirect movements, taking into account the order in which movements happen during a fixed time-period. The OCC is the number of nodes in contact with a certain node, the root, through movements of animals leaving the root. In other words, the *set of influence* of the root corresponds to the set of nodes that can be reached from the root through time-respecting paths within the observation window. Similar to the OCC, the ICC is the number of nodes in contact with the root holding through movements reaching the root. The *source set* of the root is defined as the set of nodes that can reach the root through time-respecting paths within the observation window. These two measures reflect the potential epidemic size of a disease in the network [32].

Network analyses were performed using the Igraph package in R software [33].

## 3. Results

### 3.1. Swine trade description

#### 3.1.1. Within-France movements

A total of 21,446 sites were recorded in the BDporc database, among them 97.9% were farms, 1.5% slaughterhouses and rendering plants, and 0.6% trade operators (Table 1). The number of farms decreased by 2.9% between June 2012 and December 2014.

**Table 1:** Number and proportion of sites categorised according to their major activity.

	Abbreviation	Type	Number	Percentage
**Breeding farms**	BS	Boar Station	73	0.35
	SEL	Nucleus	117	0.56
	MU	Multiplier	343	1.63
**Production farms**	PW	Post-weaning	162	0.77
	PWF	Post-weaning—Finishing	2,273	10.83
	FA	Farrowing	465	2.21
	FF	Farrowing-to-Finishing	5,064	24.12
	FPW	Farrowing—Post-weaning	288	1.37
	FI	Finishing	4,414	21.02
	SP*	Small Production	7,457	35.51
	WB	Wild-boar	342	1.63
**Total no. of farms**			**20,998**	**100**
	TR	Trade operators	117	
	SR	Slaughterhouses / Rendering plants	331	

As expected given the pyramidal structure inherent to the pig production system, PWF, FF, FPW, FI and SP are the most represented farm types in France.

* Small Production farms were defined as farms rearing fewer than 80 animals.

The database contained 2,382,510 movement records, from which 9% were discarded after the cleaning process (16, 44, and 40% due to missing or incomplete round, foreign movements or missing herd identification numbers, and animal mortality or missing animal category, respectively). A total of 838,777 rounds occurred between June 2012 and December 2014. They were composed of several loading and unloading operations: rounds between farms implied on average 2.5 holdings (range: 2–32), whereas rounds going to slaughterhouses were on average composed of a single movement. The leading destination of movements was slaughterhouses/rendering plants (75.2% of unloading operations), followed by farms (22.8%) and trade operators (2.0%). Growing pigs were the main animal category involved in movements (67% of unloaded animals), followed by piglets (31%) and breeding pigs (2%). The average number of animals transported in a given round varied with the destination site: in the second half of 2014, a round going to farms transported on average 188 animals, whereas those going to slaughterhouses and trade operators transported on average 84 and 25 pigs, respectively. The number of animals transported in a single round increased by 4%, 1.6% and 24.8% over the study period for rounds going to farms, slaughterhouses and trade operators, respectively. The number of rounds decreased by 4% over the same period, leading to an overall decrease of 0.6% in the total number of unloaded animals. The decline in exchanges mainly affected breeding pigs and trade operators. These data are detailed in S1 Table.

The distribution of distances travelled by pigs in a round varied with the animal category. Excluding movements to slaughterhouses, rendering plants and trade operators from distance calculations, breeding pigs travelled on average 270 km (median: 200, range: 0–1,000), whereas growing pigs travelled on average 74 km (median: 42, range: 0–999).

#### 3.1.2. Movements from/to foreign countries

A total of 12,065 rounds came from or went abroad over the study period, corresponding to 1.4% of the total number of rounds recorded in the whole database. Animals sent abroad were mostly growing pigs (59.4% of animals unloaded abroad), culled sows and boars (28.7%) and breeding pigs (9.6%). Outgoing shipments mainly went to Belgium and Germany (48.6% and 32.1%, respectively—mainly pigs and culled sows/boars to slaughterhouse), Italy (7.0%—mainly pigs to slaughterhouses) and Spain (7.2%—mainly pigs to slaughterhouses and breeding pigs). Animals imported from abroad were growing pigs, piglets and breeding pigs (43.6%, 38.0% and 18.1%, respectively). Incoming shipments came primarily from Spain (47.3%—mainly pigs to slaughterhouses), Belgium (33.3%—mainly piglets) and Denmark (11.5%—mainly breeding pigs). Shipments to and from non-EU countries represented only 0.5% and 0.4% of foreign movements, respectively.

### 3.2. Network description

#### 3.2.1. Network mapping

The density of active holdings and movements varied with regions, e.g. the network in north-western France was much denser than in south-eastern France (Fig 2.1). Breeding farms were mostly located in the upper left diagonal part (Fig 2.2). The network appeared denser using the TM than the AIM. Node degree was higher in the TM approach than in the AIM, especially for farrowing and farrow-to-finish farms, and particularly in the centre of France (Fig 2.3.B). Network maps were similar over the five semesters (data not shown).

**Fig 2:**
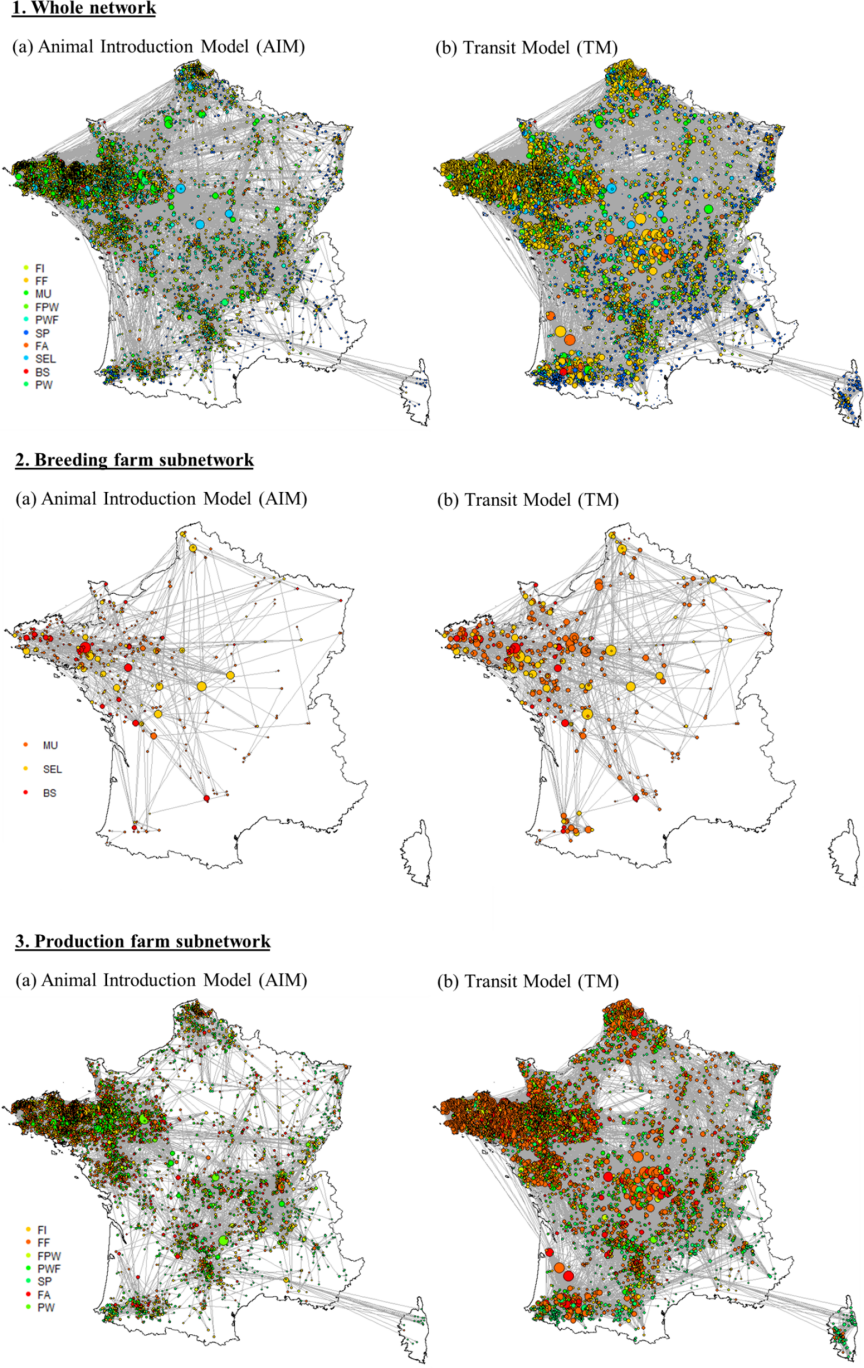
Mapping of the pig movement network in France (second half of 2014) applying the two different models (Animal Introduction Model [AIM] and Transit Model [TM]) to the whole network, the breeding farm subnetwork and the production farm subnetwork. The points are active holdings only (i.e. farms having had at least one movement over the semester). Their size is proportional to their degree. Direct movements to slaughterhouses are excluded. BS: boar station, SEL: nucleus, MU: multiplication, FA: farrowing, FF: farrowing-to-finishing, FI: finishing, FPW: farrowing-post-weaning, PW: post-weaning, PWF: post-weaning-finishing, SP: small producers.

#### 3.2.2. Network descriptive indicators

**Whole network.** Network descriptive statistics are summarised in Table 2.

**Table 2:** Descriptive indicators of the pig movement network in France (2012–2014) using the two different network models (Animal Introduction Model [AIM] and Transit Model [TM]) and for three different considered populations (whole network, breeding farm subnetwork, production farm subnetwork).

**Whole network**																				
**Semester**	**No. of active nodes**		**No. of links**		**Average degree**		**Average path length**		**Diameter**		**Density** **(x10**^**-4**^**)**		**Clustering coefficient**		**% of pairs of nodes with Jaccard similarity coefficient = 0**		**Assortativity degree**		**Reciprocity ratio**	
	**AIM**	**TM**	**AIM**	**TM**	**AIM**	**TM**	**AIM**	**TM**	**AIM**	**TM**	**AIM**	**TM**	**AIM**	**TM**	**AIM**	**TM**	**AIM**	**TM**	**AIM**	**TM**
**2012–2**	11,558	14,166	23,477	137,348	4.06	19.39	2.55	5.78	10	20	1.76	6.84	0.015	0.27	99.59	98.11	-0.079	0.19	0.0045	0.090
**2013–1**	11,419	14,161	22,969	134,901	4.02	19.05	2.28	5.78	10	20	1.76	6.73	0.015	0.26	99.60	98.12	-0.097	0.17	0.0050	0.090
**2013–2**	11,223	13,817	22,367	136,235	3.99	19.72	2.36	5.75	11	18	1.77	7.14	0.015	0.27	99.59	98.05	-0.11	0.18	0.0051	0.097
**2014–1**	11,013	13,784	21,691	132,677	3.94	19.25	2.46	5.88	9	20	1.79	6.98	0.014	0.27	99.60	98.07	-0.085	0.18	0.0061	0.093
**2014–2**	10,872	13,360	20,842	129,155	3.83	19.33	2.22	5.82	11	21	1.76	7.24	0.013	0.27	99.63	98.00	-0.11	0.20	0.0055	0.094
**Breeding farm subnetwork**																				
**Semester**	**No. of active nodes**		**No. of links**		**Average degree**		**Average path length**		**Diameter**		**Density**		**Clustering coefficient**		**% of pairs of nodes with Jaccard similarity coefficient = 0**		**Assortativity degree**		**Reciprocity ratio**	
	**AIM**	**TM**	**AIM**	**TM**	**AIM**	**TM**	**AIM**	**TM**	**AIM**	**TM**	**AIM**	**TM**	**AIM**	**TM**	**AIM**	**TM**	**AIM**	**TM**	**AIM**	**TM**
**2012–2**	404	453	703	1,826	3.48	8.06	1.78	6.16	5	20	4.31	8.92	0.035	0.30	94.97	89.64	-0.040	0.15	0.010	0.13
**2013–1**	396	446	716	1,879	3.62	8.43	1.71	5.92	4	16	4.58	9.47	0.035	0.30	94.12	88.67	-0.038	0.15	0.0042	0.12
**2013–2**	395	452	648	1,796	3.28	7.95	1.73	7.18	4	21	4.16	8.81	0.031	0.33	95.33	90.86	-0.029	0.18	0.014	0.13
**2014–1**	401	454	654	1,806	3.26	7.96	1.78	7.56	5	23	4.08	8.78	0.029	0.32	95.25	90.61	-0.047	0.09	0.011	0.13
**2014–2**	392	445	616	1,753	3.14	7.88	1.73	6.99	5	22	4.02	8.87	0.034	0.35	95.52	90.70	-0.039	0.17	0.0065	0.12
**Production farm subnetwork**																				
**Semester**	**No. of active nodes**		**No. of links**		**Average degree**		**Average path length**		**Diameter**		**Density**		**Clustering coefficient**		**% of pairs of nodes with Jaccard similarity coefficient = 0**		**Assortativity degree**		**Reciprocity ratio**	
	**AIM**	**TM**	**AIM**	**TM**	**AIM**	**TM**	**AIM**	**TM**	**AIM**	**TM**	**AIM**	**TM**	**AIM**	**TM**	**AIM**	**TM**	**AIM**	**TM**	**AIM**	**TM**
**2012–2**	9,730	12,653	14,243	119,657	2.93	18.91	2.01	6.21	8	23	1.50	7.47	0.019	0.28	99.82	98.18	-0.058	0.27	0.0068	0.10
**2013–1**	9,561	12,559	13,742	116,842	2.87	18.61	1.64	6.20	7	21	1.50	7.41	0.021	0.27	99.83	98.19	-0.056	0.26	0.0078	0.10
**2013–2**	9,340	12,193	13,538	118,333	2.90	19.41	1.64	6.09	7	20	1.55	7.96	0.020	0.28	99.83	98.08	-0.017	0.26	0.0074	0.11
**2014–1**	9,130	12,053	12,895	115,194	2.82	19.11	1.60	6.27	7	22	1.55	7.93	0.017	0.28	99.83	98.06	-0.00074	0.26	0.0089	0.10
**2014–2**	8,955	11,820	12,675	111,939	2.83	18.94	1.64	6.22	10	21	1.58	8.01	0.016	0.28	99.82	98.01	-0.019	0.28	0.0083	0.10

**Fig 3:**
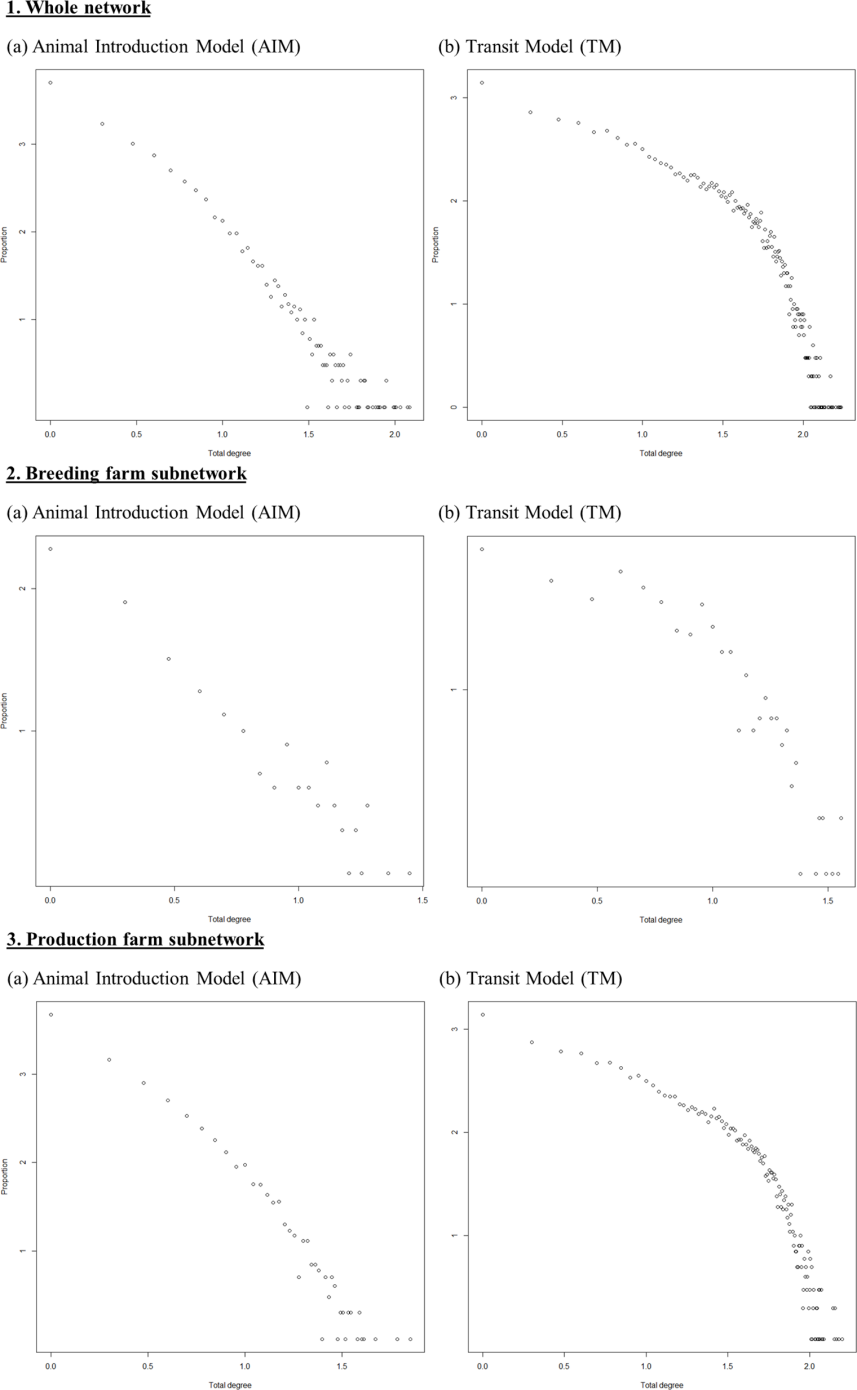
Distribution of pig farm degrees (log scale) using the two different network models (Animal Introduction Model [AIM] and Transit Model [TM]) and in three different considered populations (whole network, breeding farm subnetwork, production farm subnetwork) (second half of 2014).

In the second half of 2014 for example, the network contained 11,013 and 13,784 active holdings when using the AIM and the TM, respectively. The number of links per semester was around six times higher in the TM than in the AIM (132,677 and 21,691 links, respectively). Regarding link multiplicity, 51% of links between two holdings happened only once per semester in the AIM versus 68% in the TM. A holding exchanged animals on average with four different farms in the AIM, while a holding was in contact with 19 other farms on average in the TM (average degree). Fig 3 shows the degree distributions of holdings on a log–log scale for the AIM and the TM. Whatever the model, the distribution appeared similar in the five semesters (data not shown) and showed power-law-like behaviour (power-law exponent *alpha* values being equal to 2.78 and 5.82 with p-values of the KS test being 0.29 and 0.78 for the AIM and the TM, respectively), suggesting a scale-free structure of the network.

Distance indicators varied with the model used: a given pair of connected nodes was separated by approximately two animal movements in the AIM versus six movements in the TM (average path length). The average path length was shorter in the AIM and similar in the TM to in a random graph of the same size. The diameter also increased from 10 links in the AIM to 20 links in the TM. The network modelled with the TM was four times denser than the AIM one. The clustering coefficients of the network were low, but ten times higher in the TM than in the AIM, suggesting that nodes tended to gather when considering the TM. Moreover, the clustering coefficient was higher in the AIM and the TM than in a random graph of the same size. Whatever the model, the Jaccard similarity coefficient was equal to zero for almost all pairs of nodes, showing the dissimilarity of nodes. The assortativity of the AIM network was negative (i.e. the network was disassortative). On the contrary, the assortativity degree of the TM network was positive, indicating that nodes were more often linked to nodes with similar degrees. Whatever the model, the reciprocity ratio was low, reflecting that links were rarely bidirectional. All these indicators were globally stable over time, at a semester scale.

**Fig 4:**
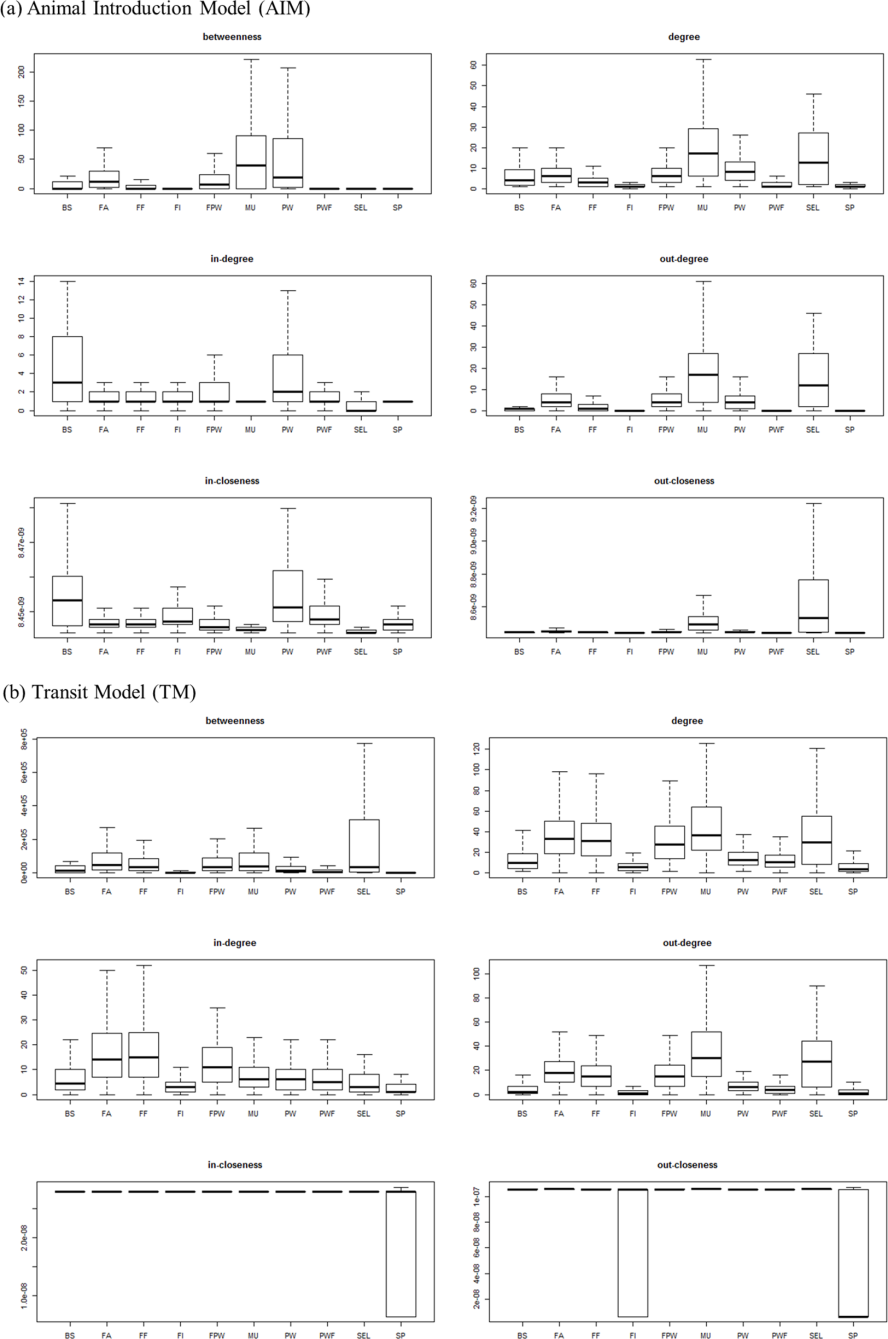
Distribution of degree, betweenness and closeness centralities of pig holdings in France according to different farm categories (second half of 2014) using the two different network models (Animal Introduction Model [AIM] and Transit Model [TM]). BS: boar station, SEL: nucleus, MU: multiplication, FA: farrowing, FF: farrowing-to-finishing, FI: finishing, FPW: farrowing-post-weaning, PW: post-weaning, PWF: post-weaning-finishing, SP: small producers.

**Specificities of breeding/production farms.** The modelling approach was found to affect more the indicators of the production farm subnetwork than the ones of the breeding farm subnetwork (Table 2). For example, comparing the TM and AIM approaches, the number of links in the production farm subnetwork was increased by a factor of eight, while it was only three-times higher in the breeding farm subnetwork. Centrality values within farm type were highly heterogeneous (Fig 4): for example, degree centrality ranged from 1 to 121 (median: 17) for multiplication farms in the AIM. For the two types of models, there were significant differences in the centrality values (degree, closeness and betweenness) between types of pig farms (Kruskal-Wallis test: *p*-value < 0.0001). In the AIM, nucleus, multiplication and post-weaning farms had higher values for degree and betweenness centrality, whereas farrowing and farrow-to-finish herds presented higher values for in-degree centrality in the TM (Fig 4).

#### 3.2.3 Detection of connected components and communities

**Connected components.** In both models, few weakly connected components (WCCs) were detected, the largest one gathering around 90% of holdings (Table 3). In the whole network, the number of WCCs increased by four times between the AIM and the TM, whereas it decreased by a factor of 1.5 in the breeding farm subnetwork, and increased by a factor of 14 in the production farm subnetwork. In the AIM, a high number of strongly connected components (SCCs) was found, the largest one containing less than 1% of farms. On the contrary, the TM network was less fragmented, with a lower number of SCCs and the detection of a giant SCC (GSCC) containing more than 70% of pig herds. The TM production farm network was more cohesive than the TM breeding farm one. Removing all farrow-to-finish herds from the production farm network led to a decrease in the size of the GSCC from 70% to 30% of the nodes contained in the GSCC. All connected components were globally stable over time, at a semester scale.

**Table 3:** Connected components in the pig movement network in France (2012–2014) using the two different network models (Animal Introduction Model [AIM] and Transit Model [TM]) and in three different considered populations (whole network, breeding farm subnetwork, production farm subnetwork).

**Whole network**								
**Semester**	**Weakly connected components (WCCs)**				**Strongly connected components (SCCs)**			
	**No. of WCCs**		**Size of largest WCC** **(% of active nodes)**		**No. of SCCs**		**Size of largest SCC** **(% of active nodes)**	
	**AIM**	**TM**	**AIM**	**TM**	**AIM**	**TM**	**AIM**	**TM**
**2012–2**	226	995	10,885 (94.2%)	13,063 (92.2%)	11,436	4,006	18 (0.2%)	10,075 (71.1%)
**2013–1**	227	1,091	10,703 (93.7%)	12,970 (91.6%)	11,290	4,087	19 (0.2%)	9,954 (70.3%)
**2013–2**	211	1,113	10,510 (93.7%)	12,629 (91.4%)	11,089	3,990	24 (0.2%)	9,700 (70.2%)
**2014–1**	232	1,207	10,261 (93.2%)	12,511 (90.8%)	10,871	4,092	17 (0.2%)	9,542 (69.2%)
**2014–2**	220	1,045	10,156 (93.4%)	12,182 (91.2%)	10,746	3,851	22 (0.2%)	9,381 (70.2%)
**Breeding farm subnetwork**								
**Semester**	**Weakly connected components (WCCs)**				**Strongly connected components (SCCs)**			
	**No. of WCCs**		**Size of largest WCC** **(% of active nodes)**		**No. of SCCs**		**Size of largest SCC** **(% of active nodes)**	
	**AIM**	**TM**	**AIM**	**TM**	**AIM**	**TM**	**AIM**	**TM**
**2012–2**	9	6	387 (95.8%)	439 (96.9%)	396	170	3 (0.7%)	254 (56.1%)
**2013–1**	7	3	382 (96.5%)	441 (98.9%)	393	197	2 (0.5%)	223 (50.0%)
**2013–2**	14	5	360 (91.14%)	444 (98.2%)	385	174	6 (1.5%)	255 (56.4%)
**2014–1**	12	5	375 (93.5%)	445 (98.0%)	394	174	3 (0.7%)	242 (53.3%)
**2014–2**	20	6	321 (81.9%)	435 (97.8%)	388	216	2 (0.5%)	178 (40.0%)
**Production farm subnetwork**								
**Semester**	**Weakly connected components (WCCs)**				**Strongly connected components (SCCs)**			
	**No. of WCCs**		**Size of largest WCC** **(% of active nodes)**		**No. of SCCs**		**Size of largest SCC** **(% of active nodes)**	
	**AIM**	**TM**	**AIM**	**TM**	**AIM**	**TM**	**AIM**	**TM**
**2012–2**	810	59	7,222 (74.2%)	12,450 (98.4%)	9,623	3,086	18 (0.2%)	9,475 (74.9%)
**2013–1**	817	65	6,888 (72.0%)	12,385 (98.6%)	9,443	3,040	19 (0.2%)	9,398 (74.8%)
**2013–2**	844	60	6,546 (70.1%)	12,046 (98.8%)	9,224	2,930	24 (0.3%)	9,130 (74.9%)
**2014–1**	861	61	6,199 (67.9%)	11,912 (98.8%)	9,008	2,902	17 (0.2%)	9,001 (74.7%)
**2014–2**	839	80	6,120 (68.3%)	11,593 (98.1%)	8,838	2,869	22 (0.2%)	8,826 (74.7%)

**Fig 5:**
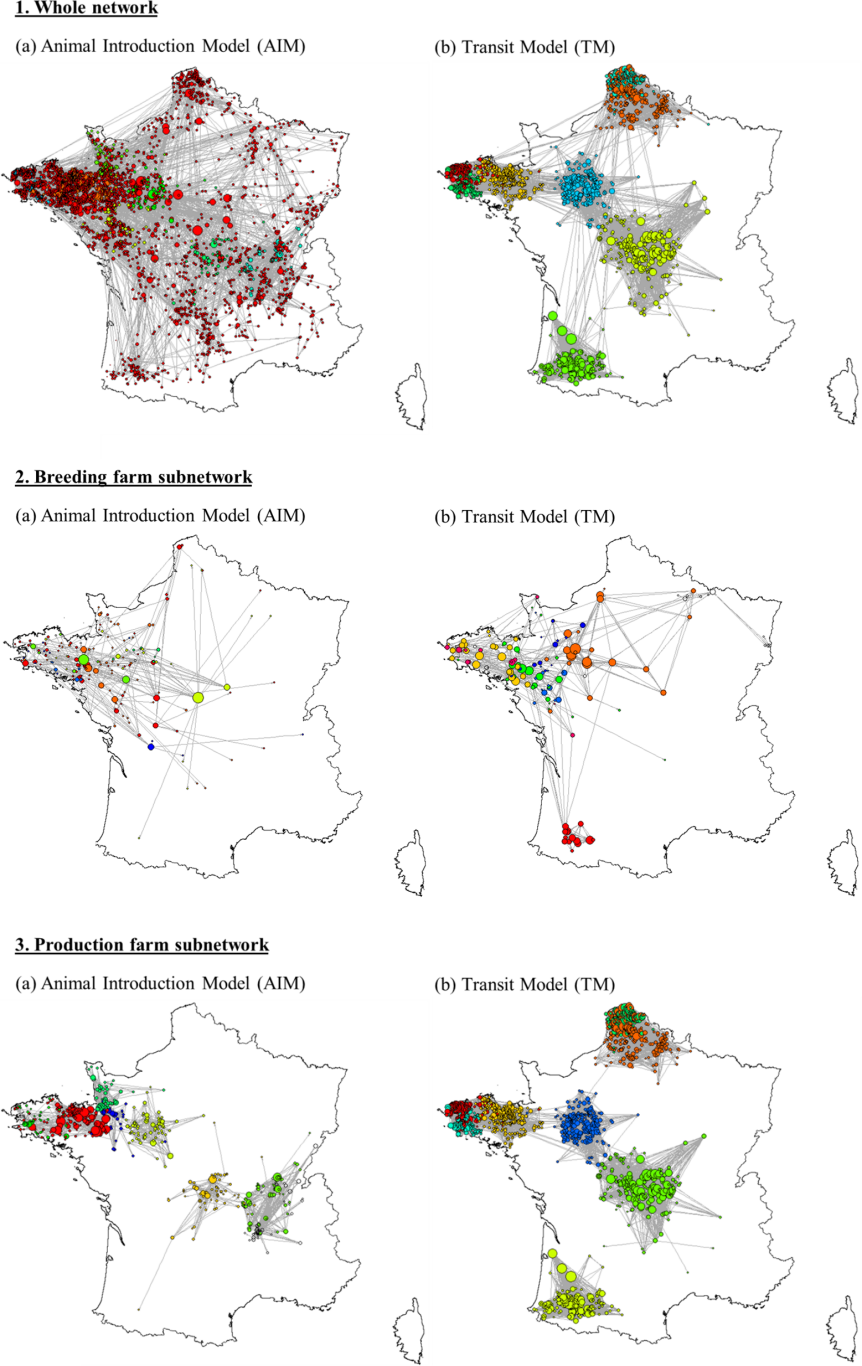
Mapping of the eight largest communities in the pig movement network in France (second half of 2014) using the two different network models (Animal Introduction Model [AIM] and Transit Model [TM]) and in three different considered populations (whole network, breeding farm subnetwork, production farm subnetwork).

**Communities.** The topology of network communities varied with the modelling assumptions. In the AIM approach, a huge geographically dispersed community was found in the whole network, whereas the TM highlighted several small geographically clustered communities (Fig 5).

In the breeding farm subnetwork, a similar number of communities was detected using the two different models, but breeding pig communities were geographically more dispersed and contained approximately four times more holdings in the AIM than in the TM (Table 4). In the production farm subnetwork, more communities were detected in the AIM than in the TM, and they gathered twice more farms. Communities were found to be permeable, since at least 25% of links connected two communities (Table 4). Communities were also found to be stable over the five semesters (maps not shown).

**Table 4:** Communities in the pig movement network in France (2012–2014) using the two different network models (Animal Introduction Model [AIM] and Transit Model [TM]) and in three different concerned populations (whole network, breeding farm subnetwork, production farm subnetwork).

**Whole network**						
**Semester**	**No. of communities**		**Size of largest community** **(% of active nodes)**		**No. of crossing links** **(% of total no. of links)**	
	**AIM**	**TM**	**AIM**	**TM**	**AIM**	**TM**
**2012–2**	1,673	1,816	3,079 (26.6%)	417 (2.9%)	9,541 (40.6%)	47,143 (34.3%)
**2013–1**	1,653	1,937	3,283 (28.8%)	384 (2.7%)	9,249 (40.3%)	45,980 (34.1%)
**2013–2**	1,573	1,957	3,344 (29.8%)	393 (2.8%)	8,758 (39.2%)	45,241 (33.2%)
**2014–1**	1,553	2,073	3,326 (30.2%)	363 (2.6%)	8,511 (39.2%)	43,628 (32.9%)
**2014–2**	1,523	1,874	3,338 (30.7%)	351 (2.6%)	8,013 (38.4%)	43,289 (33.5%)
**Breeding farm subnetwork**						
**Semester**	**No. of communities**		**Size of largest community** **(% of active nodes)**		**No. of crossing links** **(% of total no. of links)**	
	**AIM**	**TM**	**AIM**	**TM**	**AIM**	**TM**
**2012–2**	73	70	81 (20.0%)	21 (4.6%)	303 (43.1%)	857 (46.9%)
**2013–1**	60	72	162 (40.9%)	32 (7.2%)	311 (43.4%)	831 (44.2%)
**2013–2**	66	71	152 (38.5%)	37 (8.2%)	236 (36.4%)	682 (38.0%)
**2014–1**	66	71	174 (43.4%)	21 (4.6%)	239 (36.5%)	739 (40.9%)
**2014–2**	75	66	66 (16.8%)	31 (7.0%)	254 (41.2%)	645 (36.8%)
**Production farm subnetwork**						
**Semester**	**No. of communities**		**Size of largest community** **(% of active nodes)**		**No. of crossing links** **(% of total no. of links)**	
	**AIM**	**TM**	**AIM**	**TM**	**AIM**	**TM**
**2012–2**	1,802	825	123 (1.3%)	407 (3.2%)	3,999 (28.1%)	38,452 (32.1%)
**2013–1**	1,787	863	178 (1.9%)	388 (3.1%)	3,655 (26.6%)	37,007 (31.7%)
**2013–2**	1,705	848	175 (1.9%)	337 (2.8%)	3,420 (25.3%)	37,181 (31.4%)
**2014–1**	1,684	872	136 (1.5%)	351 (2.9%)	3,335 (25.9%)	35,625 (30.9%)
**2014–2**	1,653	874	181 (2.0%)	335 (2.8%)	3,217 (25.4%)	34,996 (31.3%)

#### 3.2.4. Temporal network analysis

**Link and node preservation.** More than 98% and 77% of nodes remained active during two consecutive semesters in the AIM and in the TM, respectively. Most holdings that were not active from one semester to another were small producers. Only 51% and 36% of links were preserved from one semester to another in the AIM and in the TM, respectively.

**Fig 6:**
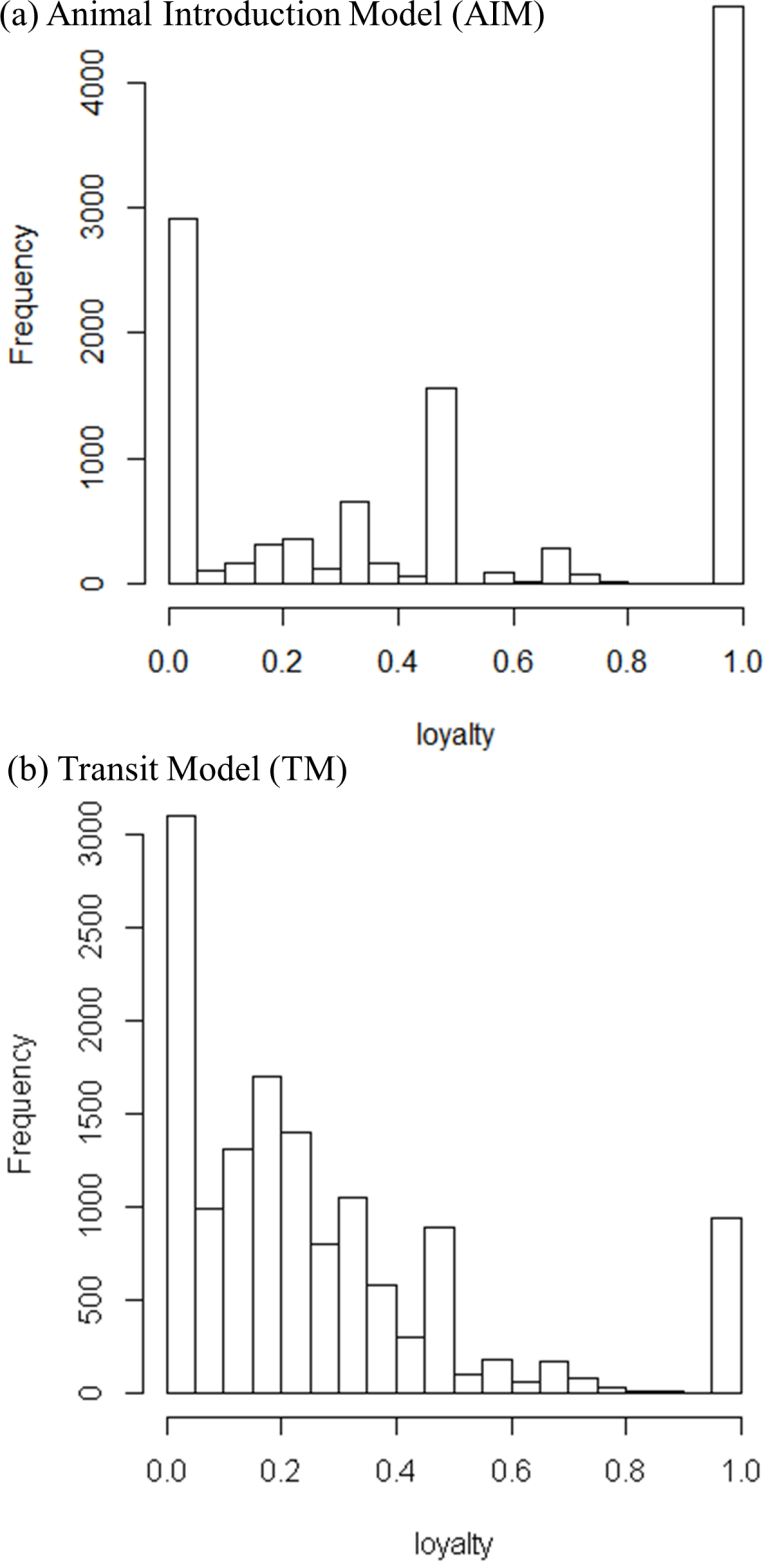
Node loyalty distributions in the pig movement network in France (second half of 2012 / first half of 2013) using the two different network models (Animal Introduction Model [AIM] and Transit Model [TM]).

**Node loyalty.** The distribution of loyalty values computed in the AIM showed two peaks in 0 and 1, whereas the TM loyalty distribution was skewed to the right (Fig 6). In both cases, the distributions reflected a diverse range of patterns between establishing new connections versus repeating existing ones. The distributions of loyalty values did not exhibit variation moving along consecutive time windows (data not shown). The 0 and 1 loyalty values corresponded to low degree nodes for which few loyalty values are available, given the loyalty definition. Node degree and node loyalty were found to be correlated in both network models (Pearson correlation coefficient *p*-value < 0.001).

**Fig 7:**
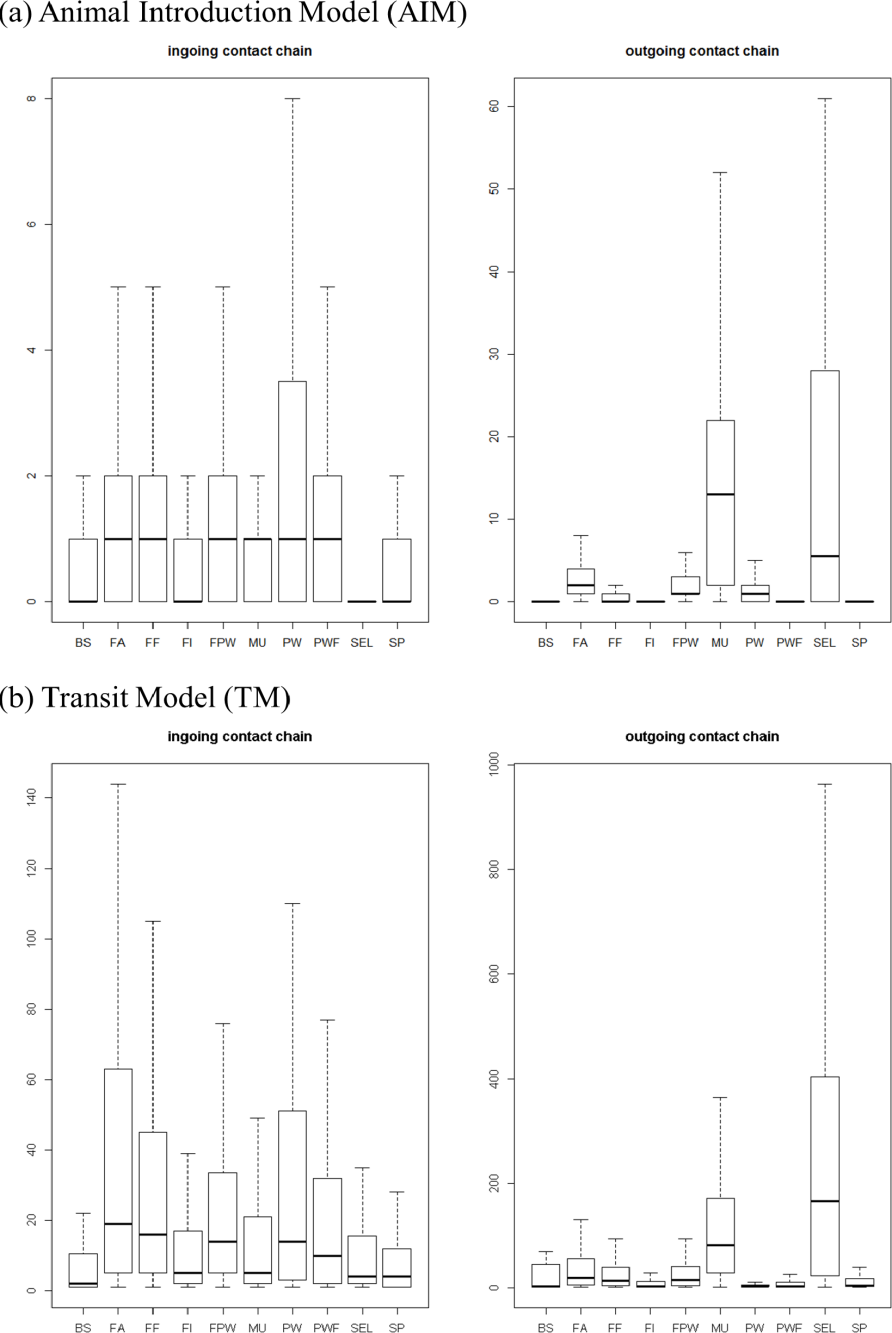
Distribution of ingoing and outgoing contact chains of pig holdings in France according to different farm categories (1 to 31 December 2014) using the two different network models (Animal Introduction Model [AIM] and Transit Model [TM]). BS: boar station, SEL: nucleus, MU: multiplication, FA: farrowing, FF: farrowing-to-finishing, FI: finishing, FPW: farrowing-post-weaning, PW: post-weaning, PWF: post-weaning-finishing, SP: small producers.

**Ingoing and outgoing contact chains.** Ingoing and outgoing contact chains computed over a one-month period exhibited different distributions depending on the network model and the farm type (Fig 7). The TM contact chain figures were much higher than the AIM ones. In the AIM and in the TM, nucleus and multiplication farms showed a larger OCC than other farm types. In the TM, the ICC was found to be higher for production farms than for the other holding types. The contact chain distributions computed over one-month periods were stable over time (data not shown).

## 4. Discussion

Exploring the topology of animal movements provides insights into disease epidemiology and gives the opportunity to implement targeted surveillance strategies and control measures. The primary interest of our study lies in building pig movement network models adapted to the epidemiological features of pathogens, in particular to their transmission route. To our knowledge, most studies in the literature only took into account direct movements of animal introduction or built two-mode networks that cannot be explored as deeply as one-mode ones [2, 6, 8, 9, 21, 27]. Only a few studies mentioned the role of trucks, material, visitors or staff as potential indirect vectors, or explored the issue of shared trucks [19, 21]. Truck transit movements may nevertheless play a central role in the transmission of highly contagious diseases such as ASF, PED, and FMD. The pig production sector is organised in a pyramidal way: at the top, nucleus farms provide purebred sows and boars to multiplication farms, which produce crossbred pigs and gilts to supply production farms, producing pigs for slaughter. Assuming that this specific structure leads to a particular topology of the movement network, we performed a multi-scale analysis suiting the pyramidal organisation: we analysed both the whole network and two subnetworks containing (1) only the breeding farms (nucleus, multipliers, and boar stations); and (2) only the production farms (from farrowers to finishers). Our network analysis did not account for movements to slaughterhouses, as they are considered as an epidemiological dead-end. Because several studies have proven that trade in the pig production sector does not show any seasonal pattern in France [2, 7, 23, 27, 28], we analysed the network at a semester scale. This temporal scale was considered appropriate to reflect the global trade behaviour of farms while making it possible to observe evolutions over the study period. Our two models were applied to all movement data recorded in France from June 2012 to December 2014 in the National Swine Identification Database (BDporc). The information provided by this database is managed by swine industry professionals, is recognised by the French Ministry for Agriculture, and can therefore be considered trustworthy. Moreover, a thorough cleaning stage was carried out to manage incorrect or incomplete data. This kind of electronic data is also more accurate than movements reported in questionnaires [34]. An even more accurate alternative would be to use GPS (Global Positioning System) to geographically locate trucks and precisely track their movements, but this would require the approval of transportation operators to share this kind of data, as well as advanced analytical methods to manage such data. In contrast with other studies that were limited to a single region or a sample of voluntary farms or to a short period of time [6, 9, 21], we used recent data from the whole country and covering a long period of time. Finally, the quality of data–in terms of accuracy, reliability, and comprehensiveness–guarantees the robustness of our results.

The analysis of movements over the study period showed a decline in the number of rounds, while the number of animals moved per round increased, leading to an overall slight decrease in animal trade movements, which was also reported in other European studies [8]. This is consistent with the intensification of the pig production industry (that is to say a decrease in the number of pig farms balanced by an increase in the number of animals reared), resulting in the observation of fewer movements involving larger pig batches. The level of round complexity was highly heterogeneous, the average number of holdings implied in a round being 2.5 but reaching 32. This is consistent with the distance travelled by pigs in a round (excluding foreign movements), ranging from 0 to 1,000 km. The distances reported in our study are longer than in other European countries such as Belgium or England/Wales [8, 19], in accordance with the results of the comparative study conducted by Relun et al. [23]. The longest and most complex rounds implied culled boar/sows and breeding pigs. They were mainly located in central and south-western France where the production is less intensive and rounds are thus composed of several movements of small batches. Movements from/to foreign countries represent a small fraction of the pig trade in France and are linked to specific markets, but they are nevertheless important to take into consideration in order to prevent the introduction of a disease that is absent from France (e.g. FMD, ASF, PED).

Whatever the modelling approach, network structure properties exhibited overall stability over the study period: *(i)* at a semester scale, active nodes globally remained the same from one semester to another, except for small production farms; *(ii)* network metrics were similar from one semester to another; and *(iii)* connected components and communities were also stable over the study period. This stability of the pig production network has already been described in several papers [8, 23, 27] and enables us to generalise the findings of our study to the current swine trade network. However, loyalty distributions showed relative volatility of farms’ trade partners, indicating that future links may be difficult to predict. The same trend has already been described in a cattle movement network [35].

Our two network models exhibited two classical patterns of connectivity described in other studies [2, 6, 8, 9, 21, 23, 28], known as *(i)* small-world, and *(ii)* scale-free topologies. *(i)* Whatever the model, our networks had higher clustering coefficients and shorter or similar average path length than random graphs of the same size (corresponding to a small-world topology) [36, 37]. This means that most nodes are not directly connected to each other but can be reached through a small number of connections. This allows diseases to spread quickly within clusters but also to reach other clusters in the network by crossing a few links. This topology facilitates persistent infection in the pig population but the size of an epidemic in a small-world network tends to be smaller when compared to a random network. *(ii)* The holdings’ degree in both networks showed power-law-like behaviour (heavy tailed distribution), meaning that many of the nodes had few connections while a few nodes had many connections (corresponding to a scale-free structure) [38]. This indicates the presence of highly connected nodes, i.e. of hubs, that are of central importance with regard to disease spread (also called super-spreaders). Epidemics can therefore spread faster in scale-free networks than in random ones. Scale-free networks can withstand random attacks but are highly vulnerable to targeted attacks towards the hubs [11, 39, 40].

Size, degree and distance metrics (average path length, diameter, density) observed in the AIM are consistent with the literature data, especially for the pig movement networks in France [23, 41]. As expected, given the model assumptions, these values increased when switching from the AIM to the TM. The differential modelling approach affected more production farms than breeding farms, suggesting that production farms may play a key role in the spread of indirectly transmitted diseases. The assortativity degree of the AIM was negative, in accordance with the results of previous studies [6, 7, 10]. However, the TM network was found to be assortative. According to [42], disassortative networks are particularly sensitive to the removal of high-degree farms since they are dispersed over the whole network. Thus, fewer holdings have to be removed to destroy the largest component compared to a network with positive assortativity degree. Like in Thakur et al. [21], the reciprocity ratio was very low, reflecting the pyramidal structure of the pig production sector with unidirectional links going from the top breeding farms to the bottom production farms. Similarly, the Jaccard similarity coefficient was zero for almost all pairs of nodes, showing that movements occurred mainly between different farm types.

Centrality values within a farm type were highly heterogeneous (except for closeness centrality, see below). In the AIM network, the high out-degree distributions observed for breeding farms compared with production farms is in accordance with previously published papers [6, 10, 21] and with the pyramidal structure of pig production. It shows their potential key role in disease spread to the whole network in case of introduction of the disease to this kind of farm. Their high betweenness score also proves that disease surveillance should be primarily directed towards these units. Indeed, holdings with a high betweenness centrality could build so-called bridges between different network components. Removing these specific holdings would fragment the network. In the TM network, farrow and farrow-to-finish farms exhibited high in-degree distribution, whereas post-weaners had the highest in-degree values in the AIM. This results in a similar total degree for farrow, farrow-to-finish, nucleus and multiplication farms in the TM. This could be explained by the fact that farrow and farrow-to-finish farms were part of more complex rounds involving more truck transit movements. It shows that farrow and farrow-to-finish farms are more vulnerable to the introduction of diseases for which indirect transmission can occur, and that surveillance measures specific to these diseases should target these farm categories. In the AIM, post-weaning and post-weaning—finishing farms exhibited the highest median ingoing closeness, which is consistent with the literature [10]. A high value for ingoing closeness centrality implies that the trade partners of a specific holding can reach the node in only a few movements. In the AIM, nucleus and multiplication farms had the highest median outgoing closeness [10]. High outgoing closeness means that a seller reaches its client in only a few steps. Thus, holdings with high outgoing closeness centrality can spread a pathogen in the production network faster. The distributions of the ingoing and outgoing closeness centralities were not highly informative in the TM because their range was too small. As explained in [43], the small range of closeness values implies that slight changes in the network structure greatly affect the ranking of farms according to the closeness centrality. Being used as additional information to the more powerful centrality parameters (see above) [10], closeness centrality is therefore not considered as the most appropriate measure for the detection of central holdings in a trade network, especially in terms of animal disease control and risk-based surveillance.

In both models, few WCCs were observed, the largest one containing around 90% of farms. This is consistent with the literature [6, 21, 27]. Like in previously published papers [6, 28], the AIM exhibited a high number of small SCCs, the largest one containing only 1% of farms. On the contrary, the TM network was less fragmented, with a low number of SCCs and the presence of a giant SCC joining 70% of farms. This is consistent with the clustering coefficient being ten times higher in the TM than in the AIM, reflecting a gathering trend. The GSCC disappeared when removing farrow and farrow-to-finish farms, showing their central role in TM network cohesion.

Community structures in networks are densely connected subgroups of nodes. Identification of communities in a trade network shows which holdings are preferentially linked. We looked for communities in both models of the swine trade network thanks to the Infomap algorithm. To our knowledge, this method has never been used in previous papers studying animal movements, although it is the only one applicable to directed networks and considered one of the best in terms of performance [30, 31]. The topology of the detected communities varied with the modelling approach: in the AIM, we detected one huge geographically dispersed community, while the TM exhibited several small geographically clustered communities. The topology of communities detected in the AIM is rather consistent with the literature, reporting communities forming spatial clusters and tending to cover quite large areas [6, 8, 23]. When considering the two subnetworks, the AIM breeding farm subnetwork presented larger communities than the TM one, whereas the AIM production farm subnetwork contained smaller communities than the TM one. Although these communities are permeable and crossing links can act as potential bridges for disease spread from one community to another, community borders could be used to define geographical compartments. Compartmentalisation can be an effective strategy for controlling disease epidemics while minimising disruption to trade business [8, 23]. Stopping disease spread within a community would reduce the probability of pathogen transfer to a connected community. Our results show that geographical compartmentalisation would be easier to limit the introduction of a disease transmitted through the indirect route than for a disease transmitted through animal introduction.

Timely movement tracking is of major interest to understand the origin of the pathogen introduction and the potential spread through downstream contacts. This is the reason why ingoing and outgoing contact chains were computed. The choice of a one-month duration period reflects the time needed to detect the occurrence of a disease and has been discussed in several papers [21, 27]. As expected, the ICC and OCC values were much higher in the TM than in the AIM, showing that the potential epidemic size would be larger for an indirectly transmitted disease than for a directly transmitted pathogen. Moreover, the AIM OCC was higher for breeding farms than for production ones, in line with their key role in the spread of a directly transmitted disease. In the TM, the ICC was higher for production farms, showing their vulnerability to indirectly transmitted disease. These results are in accordance with the other centrality measures (see above) and, for the AIM, with previously published papers [21, 27].

## 5. Conclusion

The primary interest of our study lies in developing, analysing and comparing two one-mode pig trade network models matching the transmission route of pathogens. From a modelling point of view, our data could be used to parametrise other models, such as exponential random graph models (ERGMs) aiming at explaining network structure [23, 44]. Our network models could also be coupled with epidemiological models of pathogen transmission within herds, this combination resulting in a between-herd epidemiological model. This kind of model would be particularly useful to understand or to assess the persistence and/or spread of a disease in a production sector. From a more operational perspective, our network models have produced useful outputs that can help to design risk-based disease surveillance and control programmes adapted to disease characteristics. They bring to light the relevance of accounting for transit movements to understand the indirect transmission of diseases. Depending on the epidemiological context, the potential epidemic size and the pathogen spread pattern would differ, as do the type of farming units that have to be targeted and the scale at which control measures should be implemented.

## Author contributions

MS performed network analysis and drafted the manuscript. MA cleaned the database, designed the network models and supervised network analysis. NR initiated and coordinated the project. All co-authors revised the manuscript and approved the final submitted version.

## Supporting information

S1 Table Pig movements within France from June 2012 to December 2014 at a semester scale. (DOCX)
