# Supplementary material for: Pig movements in France: Designing network models fitting the transmission route of pathogens
Source: PLoS One. 2017 Oct 19;12(10):e0185858. doi: 10.1371/journal.pone.0185858 (PMC5648108; doi:10.1371/journal.pone.0185858)
Supplement: S1 Table — (DOCX) [file pone.0185858.s001.docx]

**S1 Table: Pig movements within France from June 2012 to December 2014 at a semester scale.**

| **Site category** | **Animal category** | **Loading operations (outgoing shipments)** | | | | | | | | | | | | | | | | | | | | | | | | | | | |
| --- | --- | --- | --- | --- | --- | --- | --- | --- | --- | --- | --- | --- | --- | --- | --- | --- | --- | --- | --- | --- | --- | --- | --- | --- | --- | --- | --- | --- | --- |
|  |  | **No. of rounds^£^** | | | | | | | | | **No. of loading operations** | | | | | | | | | **No. of loaded animals** | | | | | | | | | |
|  |  | 2012-2 | 2013-1 | | 2013-2 | | 2014-1 | | 2014-2 | | 2012-2 | 2013-1 | | 2013-2 | | 2014-1 | | 2014-2 | | 2012-2 | | 2013-1 | | 2013-2 | | 2014-1 | | 2014-2 | |
|  |  | Number | %* | | % | | % | | % | | Number | % | | % | | % | | % | | Number | | % | | % | | % | | % | |
| **Farms** | **TOTAL for farms^$^** | 170,804 | -0.75 | | -1.79 | | -1.61 | | -3.93 | | 247,687 | -1.92 | | -2.45 | | -2.99 | | -4.80 | | 17,380,064 | | -1.30 | | 0.02 | | -1.31 | | -0.57 | |
|  | Piglets | 29,101 | -6.21 | | -1.03 | | -5.08 | | -1.32 | | 34,360 | -6.68 | | -1.88 | | -5.55 | | -1.65 | | 5,194,806 | | -2.29 | | 0.97 | | -0.27 | | 2.50 | |
|  | Growing pigs | 127,914 | 0.51 | | -1.06 | | -0.33 | | -3.15 | | 168,449 | -0.27 | | -2.01 | | -1.21 | | -4.31 | | 11,776,537 | | -0.81 | | -0.34 | | -1.63 | | -1.79 | |
|  | Breeding pigs | 22,247 | -4.14 | | -7.69 | | -7.01 | | -10.36 | | 44,878 | -4.44 | | -4.57 | | -7.73 | | -9.05 | | 408,721 | | -3.01 | | -1.80 | | -5.35 | | -4.57 | |
| **Trade operators (TR)** | **TOTAL for TR^$^** | 752 | 2.93 | | 1.86 | | -4.39 | | -30.32 | | 985 | 0.51 | | -2.74 | | -7.61 | | -27.92 | | 46,000 | | 19.13 | | 20.60 | | -6.01 | | -16.16 | |
|  | Piglets | 86 | -5.81 | | 22.09 | | 8.14 | | 46.51 | | 101 | -8.91 | | 11.88 | | -1.98 | | 31.68 | | 8,915 | | 34.12 | | 97.39 | | 28.96 | | 69.00 | |
|  | Growing pigs | 275 | 2.55 | | -18.18 | | -18.55 | | -28.00 | | 275 | 3.27 | | -14.55 | | -16.36 | | -25.82 | | 14,699 | | 28.42 | | 6.56 | | -7.94 | | -25.44 | |
|  | Breeding pigs | 569 | 1.05 | | 0.70 | | -1.41 | | -38.14 | | 609 | 0.82 | | 0.16 | | -4.60 | | -38.75 | | 22,386 | | 7.07 | | -0.75 | | -18.66 | | -43.98 | |
| **Slaughterhouses / rendering plants (SR)** | **TOTAL for SR^$^** | 50 | 28.00 | | 30.00 | | 18.00 | | -22.00 | | 50 | 28.00 | | 30.00 | | 18.00 | | -22.00 | | 3,792 | | 21.18 | | 45.83 | | 16.88 | | -17.99 | |
|  | Piglets | 0 | - | | - | | - | | - | | 0 | - | | - | | - | | - | | 0 | | - | | - | | - | | - | |
|  | Growing pigs | 3 | 0.00 | | -66.67 | | -66.67 | | -66.67 | | 3 | 0.00 | | -66.67 | | -66.67 | | -66.67 | | 100 | | -27.00 | | -78.00 | | -97.00 | | -30.00 | |
|  | Breeding pigs | 47 | 29.79 | | 31.91 | | 23.40 | | -19.15 | | 47 | 29.79 | | 31.91 | | 23.40 | | -19.15 | | 3692 | | 22.48 | | 28.22 | | 19.96 | | -17.66 | |
| **TOTAL** | **TOTAL** | 171,496 | -0.74 | | -1.79 | | -1.64 | | -4.07 | | 248,722 | -1.90 | | -2.45 | | -3.01 | | -4.89 | | 17,429,856 | | -1.24 | | 0.08 | | -1.32 | | -0.61 | |
|  | Piglets | 29,187 | -6.21 | | -0.95 | | -5.04 | | -1.18 | | 34,461 | -6.68 | | -1.83 | | -5.54 | | -1.56 | | 5,203,721 | | -2.23 | | 1.15 | | -0.22 | | 2.62 | |
|  | Growing pigs | 128,192 | 0.52 | | -1.10 | | -0.37 | | -3.21 | | 168,727 | -0.26 | | -2.03 | | -1.23 | | -4.34 | | 11,791,336 | | -0.77 | | -0.33 | | -1.64 | | -1.82 | |
|  | Breeding pigs | 22,863 | -3.94 | | -7.40 | | -6.81 | | -11.07 | | 45,534 | -4.34 | | -4.47 | | -7.66 | | -9.45 | | 434,799 | | -2.28 | | -1.49 | | -5.82 | | -6.71 | |
| **Site category** | **Animal category** | **Unloading operations (incoming shipments)** | | | | | | | | | | | | | | | | | | | | | | | | | | | |
|  |  | **No. of rounds^£^** | | | | | | | | | **No. of unloading operations** | | | | | | | | | | **No. of unloaded animals** | | | | | | | | |
|  |  | 2012-2 | | 2013-1 | | 2013-2 | | 2014-1 | | 2014-2 | 2012-2 | | 2013-1 | | 2013-2 | | 2014-1 | | 2014-2 | | 2012-2 | | 2013-1 | | 2013-2 | | 2014-1 | | 2014-2 |
|  |  | Number | | % | | % | | % | | % | Number | | % | | % | | % | | % | | Number | | % | | % | | % | | % |
| **Farms** | **TOTAL for farms^$^** | 29,381 | | -0.92 | | -0.23 | | -2.20 | | -2.39 | 44,123 | | -1.12 | | 0.01 | | -3.00 | | -3.75 | | 5,303,894 | | -1.57 | | 1.50 | | 0.15 | | 2.49 |
|  | Piglets | 22,813 | | -1.07 | | -0.05 | | -1.35 | | -2.04 | 25,597 | | -1.15 | | -0.32 | | -1.45 | | -2.45 | | 5,089,063 | | -1.62 | | 1.49 | | 0.28 | | 2.72 |
|  | Growing pigs | 519 | | 4.05 | | 2.70 | | -0.39 | | 13.10 | 528 | | 7.01 | | 4.92 | | 0.19 | | 14.02 | | 27,194 | | -5.70 | | -3.31 | | -12.18 | | -9.13 |
|  | Breeding pigs | 6,183 | | -0.68 | | -0.57 | | -5.11 | | -4.14 | 17,998 | | -1.32 | | 0.33 | | -5.29 | | -6.11 | | 187,637 | | 0.44 | | 2.34 | | -1.75 | | -2.10 |
| **Trade operators (TR)** | **TOTAL for TR^$^** | 4,722 | | -4.81 | | -19.34 | | -8.77 | | -31.89 | 5,126 | | -4.49 | | -18.73 | | -7.65 | | -28.13 | | 98,708 | | 6.07 | | -8.13 | | -7.84 | | -11.38 |
|  | Piglets | 119 | | 30.25 | | 15.13 | | 45.38 | | 102.52 | 133 | | 26.32 | | 9.77 | | 37.59 | | 87.97 | | 8,023 | | 50.64 | | 51.03 | | 51.10 | | 89.02 |
|  | Growing pigs | 522 | | 1.34 | | -19.16 | | -17.43 | | -21.46 | 525 | | 2.48 | | -16.76 | | -16.19 | | -19.43 | | 33,819 | | 14.40 | | -23.42 | | -25.01 | | -27.14 |
|  | Breeding pigs | 4,387 | | -5.88 | | -20.40 | | -8.41 | | -33.46 | 4,468 | | -6.22 | | -19.81 | | -7.99 | | -32.61 | | 56,866 | | -5.18 | | -7.38 | | -5.94 | | -16.18 |
| **Slaughterhouses / rendering plants (SR)** | **TOTAL for SR^$^** | 136,695 | | -0.62 | | -1.13 | | -1.07 | | -3.19 | 144,795 | | -1.17 | | -1.13 | | -1.41 | | -3.05 | | 11,885,222 | | -1.08 | | -0.27 | | -1.78 | | -1.54 |
|  | Piglets | 6,174 | | -26.97 | | -3.82 | | -19.60 | | 0.89 | 6,189 | | -27.06 | | -3.91 | | -19.63 | | 0.79 | | 93,654 | | -36.08 | | -26.92 | | -32.01 | | -7.45 |
|  | Growing pigs | 126,630 | | 0.34 | | -0.86 | | -0.32 | | -3.12 | 126,977 | | 0.37 | | -0.70 | | -0.15 | | -3.09 | | 11,635,291 | | -0.78 | | -0.09 | | -1.50 | | -1.48 |
|  | Breeding pigs | 11,627 | | -4.22 | | -4.28 | | -5.51 | | -4.61 | 11,629 | | -4.22 | | -4.30 | | -5.53 | | -4.63 | | 156,277 | | -2.28 | | 2.64 | | -4.31 | | -2.14 |
| **TOTAL** | **TOTAL** | 170,406 | | -0.81 | | -1.56 | | -1.55 | | -3.86 | 194,044 | | -1.25 | | -1.33 | | -1.94 | | -3.87 | | 17,287,824 | | -1.19 | | 0.23 | | -1.22 | | -0.36 |
|  | Piglets | 29,106 | | -6.43 | | -0.79 | | -5.03 | | -0.99 | 31,919 | | -6.06 | | -0.97 | | -4.81 | | -1.44 | | 5,190,740 | | -2.16 | | 1.06 | | -0.22 | | 2.67 |
|  | Growing pigs | 127,671 | | 0.36 | | -0.92 | | -0.39 | | -3.13 | 128,030 | | 0.40 | | -0.75 | | -0.21 | | -3.09 | | 11,696,304 | | -0.75 | | -0.17 | | -1.59 | | -1.58 |
|  | Breeding pigs | 22,197 | | -3.56 | | -6.43 | | -5.97 | | -10.18 | 34,095 | | -2.95 | | -3.89 | | -5.73 | | -9.08 | | 400,780 | | -1.42 | | 1.08 | | -3.34 | | -4.11 |

* percentage change compared to the second half of 2012

$ the total number of rounds is not equal to the sum of the number of rounds for each animal category because animals from several categories can be transported in the same round

£ Loading and unloading figures are different because of the rounds loaded in France and unloaded abroad.

TR: Trade Operators; SR: Slaughterhouses and Rendering plants
